# Distinct Temperature Trends in the Uptake of Gaseous *n*-Butylamine on Two Solid Diacids

**DOI:** 10.1021/acsestair.3c00032

**Published:** 2023-11-29

**Authors:** Yixin Li, Pascale S. J. Lakey, Michael J. Ezell, Kristen N. Johnson, Manabu Shiraiwa, Barbara J. Finlayson-Pitts

**Affiliations:** Department of Chemistry, University of California, Irvine, Irvine, California 92697-2025, United States

**Keywords:** reactive uptake, dicarboxylic acids, amines, temperature dependence, ionic liquid

## Abstract

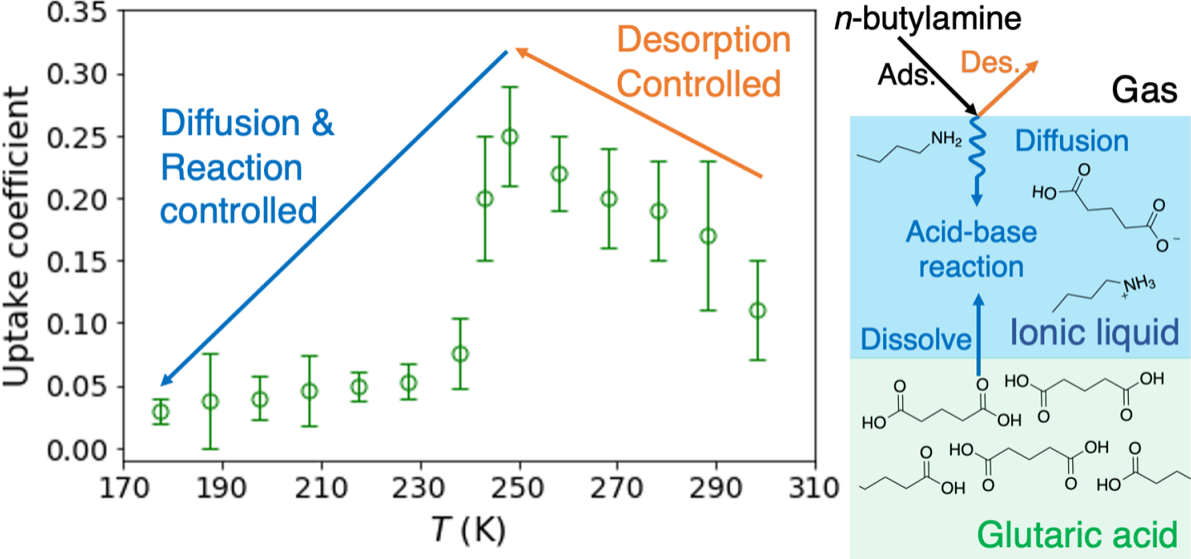

Uptake coefficients of *n-*butylamine (BA) on solid succinic (SA) and glutaric acids (GA) from 298 to 177 K were measured using a newly combined Knudsen cell temperature-programmed desorption apparatus. The uptake coefficients on SA increase monotonically from (1.9 ± 0.5) × 10^–4^ at 298 K to 0.14 ± 0.05 at 177 K (errors represent 2σ statistical errors, overall errors are estimated to be ±60%). This is consistent with a surface reaction mechanism to form solid aminium carboxylate. In contrast, the uptake coefficients on GA increase from 0.11 ± 0.04 at 298 K to 0.25 ± 0.04 at 248 K but then decrease to 0.030 ± 0.010 at 177 K. This unusual trend in temperature dependence of the uptake coefficient is due to formation of an ionic liquid (IL) layer upon the surface reaction of BA with GA, leading to a competition between the rate of desorption of BA and the rates of diffusion and reaction within the IL. Overall, the kinetic multi-layer model of aerosol surface and bulk chemistry (KM-SUB) satisfactorily reproduces these unique trends. This work provides mechanistic insight and predictive capability for the temperature-dependence of reactive uptake processes involving multiple phase changes upon surface reaction.

## Abstract 2

There are few quantitative data on reactive uptake of gases across the temperature range found in the troposphere. We report here unique temperature trends for reactive uptake of amines on two solid dicarboxylic acids, highlighting processes that will impact model predictions of particle reactivity and growth.

## 1 Introduction

Secondary organic aerosol particles (SOA), produced from oxidation of volatile organic compounds (VOCs),^1−3^ represent a major component of global fine particulate matter and have profound impacts on climate, air quality, and human health.^4−13^ These aerosol particles modify the Earth’s radiation budget directly by absorbing/scattering incoming solar radiation and indirectly by serving as cloud condensation nuclei (CCN).^14−16^ Particles larger than 100 nm reduce visibility by scattering sunlight.^16^ Ultrafine particles (< 100 nm) are able to travel deeply into the respiratory tract, exerting large adverse health effects on humans.^9−13^

The atmospheric impacts of SOA are determined by their formation and transformation processes.^14,17^ Among those processes, reactive uptake of gas species can significantly alter the physiochemical properties of particles, e.g., through changes in mass, composition, and morphology of particles, thus impacting their light scattering and absorption.^14,18,19^ Multiphase reaction products from reactive uptake may have different hygroscopicity, volatility, phase transition temperatures, and toxicity compared to the original components, altering the CCN activity, viscosity, interactions with gases, and health effects of SOA.^20−24^ Moreover, reactive uptake by aerosol particles can alter the abundance and distribution of gases, representing an important sink for many trace gases in the atmosphere.^25^

Reactive uptake of gaseous amines by solid dicarboxylic acids via an acid–base reaction to form aminium carboxylate salts is a good model system for understanding multiphase processes involving SOA.^26−29^ Amines are abundant gaseous species with a global annual budget of over ∼300 Gg N a^–1^ and are frequently detected in atmospheric particulate matter.^28^ Low molecular weight dicarboxylic acids are also ubiquitous in the atmosphere, constituting a large fraction of SOA.^30−32^ Both types of compounds, which often coexist in aerosol particles,^33−35^ are involved in new particle formation and contribute to the growth of particles by gas-particle partitioning and multiphase reactions.^36−42^

For diacids with different carbon numbers, odd–even effects are observed in their physical properties, including volatility, melting points, sublimation enthalpies, and solubilities, which is related to their solid-state structure.^32^ Previously, a series of Knudsen cell studies showed significant reactive uptake of amines and ammonia by C_3_–C_7_ dicarboxylic acids,^26,43,44^ with the uptake coefficients for odd carbon diacids being one to five orders of magnitude larger than those for even carbon diacids at room temperature.^43^ This difference was attributed to the formation of an ionic liquid (IL) surface layer for the reactions between the odd carbon diacids and amines/ammonia. The IL layer was able to dissolve underlying diacid molecules, allowing them to continuously react with amine taken up from the gas phase.^26,43^ In contrast, the reactions between the even carbon diacids and amines/ammonia formed solid salts so that diffusive processes were slower, resulting in less net reaction and smaller uptake coefficients.^26^

The temperature in the Earth’s atmosphere varies with latitude, longitude, altitude, season, and time of day.^1^ The average temperature of the troposphere typically varies from 290 to 217 K, but the lowest temperature in Antarctica at sea level can reach 184 K.^2,45,46^ This temperature variation will impact reactive uptake processes.^1,47,48^ For example, studies of the effects of temperature and phase on OH oxidation of Suwannee River fulvic acid particles showed impacts on the composition of the particles and their properties such as water uptake.^22^ Zhou et al.^49^ showed that the initial uptake coefficients of sulfur dioxide on Chinese mineral dust decreased with increasing temperature over a range from 253 to 313 K. Gao et al.^50^ studied the kinetics of the heterogeneous reaction of *n*-butylamine (BA) with succinic acid (SA) using a flow system combined with attenuated total reflection Fourier-transform infrared spectroscopy at temperatures ranging from 295 to 263 K. They reported that the uptake coefficients increased as the temperature decreased, with an effective activation energy of −71.9 kJ mol^–1^.^50^

Currently, the temperature dependence for reactive uptake processes, especially when phase changes are involved, is not well understood. A better parameterization of these processes over a wide range of temperatures is essential for accurately representing them in atmospheric models under various atmospheric conditions.^47^ In this study, a Knudsen cell in a newly designed apparatus that was combined with temperature-programmed desorption capabilities was used to measure the uptake coefficients of BA on SA (C_4_ dicarboxylic acid) and glutaric (GA, C_5_ dicarboxylic acid) acids from 177 to 298 K, spanning the temperature range of multiple phase changes. The kinetic multi-layer model of aerosol surface and bulk chemistry (KM-SUB)^51^ was applied to elucidate the processes controlling the uptake of BA on GA.

## 2 Materials and Methods

### 2.1 Instrumentation

**Figure 1:**
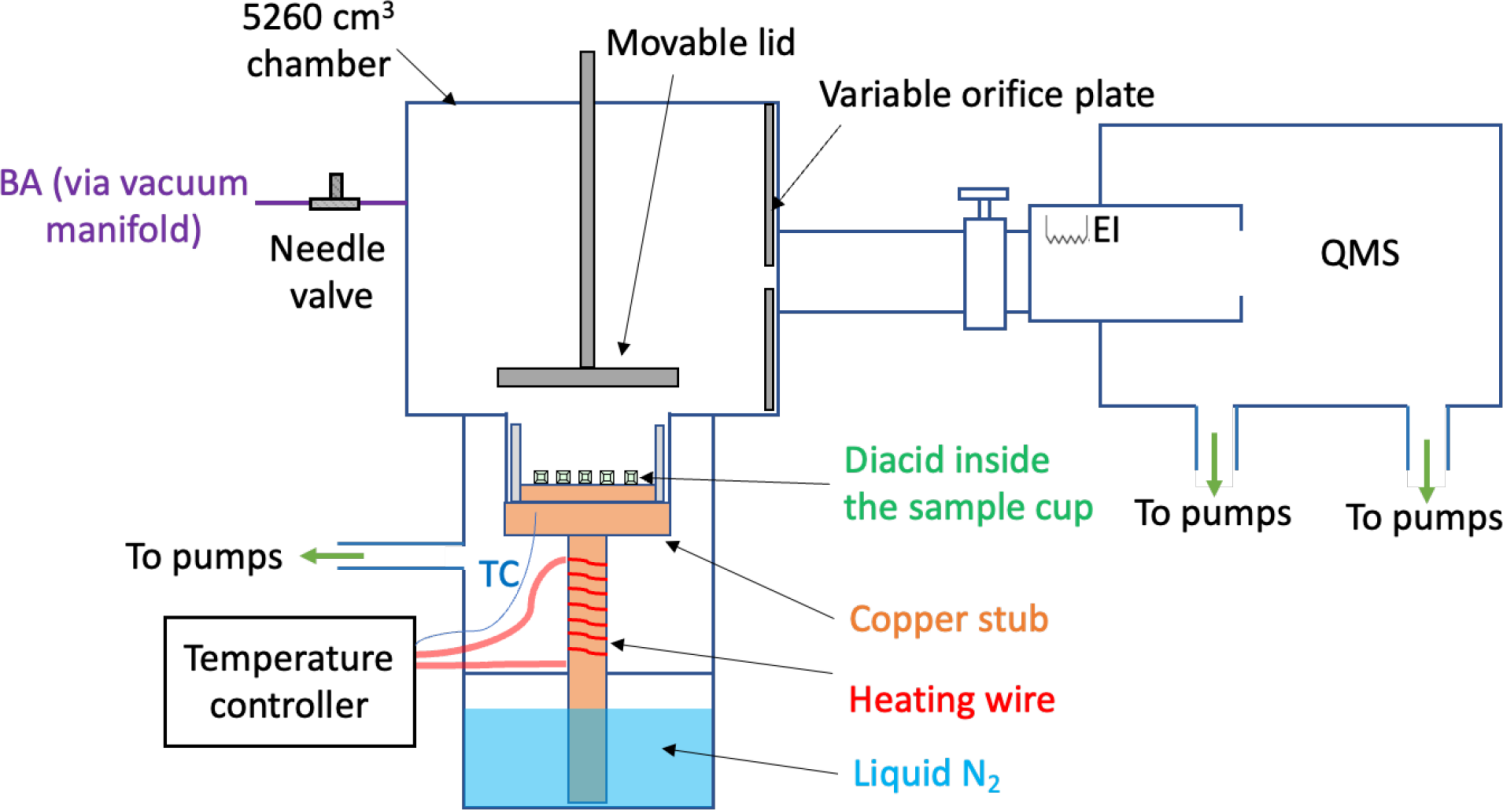
Simplified schematic of the custom-designed Knudsen cell. TC = thermocouple, QMS = quadrupole mass spectrometer, and EI = electron impact ionization source.

A custom-designed Knudsen cell where the sample temperature could be varied from 106 to 400 K (described in detail elsewhere^52^) was used to measure the temperature-dependent uptake coefficients of BA on SA and GA (Figure 1). Briefly, the diacid particles were distributed across the bottom of a sample cup that sat on a copper stub. The temperature of the stub and hence the sample was controlled by simultaneously cooling the stub with liquid nitrogen and heating using a nichrome wire connected to a temperature controller (Eurotherm 3216). The temperature was measured using a thermocouple implanted into the copper stub. The temperature difference between the copper stub and the sample was within ±1 K. Above the sample cup was a stainless-steel chamber (total volume *V* = 5260 cm^3^) whose inner surface was coated with halocarbon wax (Halocarbon Products Corporation, Series 1500) to minimize uptake and reactions on the chamber walls. The sample cup could be isolated from the chamber using a movable stainless-steel lid. The BA vapor entered the cell through a needle valve and escaped through variable orifices to a quadrupole mass spectrometer (QMS, Extrel Core Mass Spectrometer) with an electron impact ionization source. The orifices could be varied with a rotary feedthrough for effective areas of 0.33 cm^2^ or 0.011 cm^2^. The concentration of BA was monitored using the QMS at *m*/*z* = 30 for the H_2_C=NH_2_^+^ fragment.^53^

### 2.2 Experimental Procedures

GA (C_5_) (>99%, Sigma-Aldrich) was sieved using 850 μm and 500 μm mesh sizes (U.S. Standard) and the particles which passed through the 850 μm mesh, but not the 500 μm mesh, were used. Similarly, SA (C_4_) (>99%, Sigma-Aldrich) was sieved using 500 μm and 425 μm mesh sizes. The average particle sizes (assuming they are cubic with side length of *L*) were calculated from the measured mass of a measured number (>100) of particles, giving *L* = (533 ± 84) μm for GA and *L* = (445 ± 60) μm for SA (all uncertainties are ±2σ to represent a 95% confidence interval). For each experiment, the solid diacid particles were weighed (Sartorius scale model 1702) and spread across the bottom of the sample cup. The sample cup was then gently shaken to distribute the particles as evenly as possible in less than a single layer, i.e., particles did not touch, and all particles were exposed to the gas directly with five faces.^54^ The particles were then pumped (at <10^–3^ Pa) for at least 5 h to remove adsorbed water. The average surface area (*A*_1_) of a single particle exposed to BA was estimated by *A*_1_ = 5*L*^2^. The total number of particles in an experiment (*N*) was calculated from the measured total mass of diacid (*m*) and the average mass per particle, *N* = *m*/(ρ*L*^3^), where ρ is the density of the diacid (1.43 g cm^–3^ for GA and 1.56 g cm^–3^ for SA). The total surface area of the diacid sample was then calculated from *A*_s_ = *N*·*A*_*1*_.

BA vapor diluted in helium (with a ratio of about 1:5) was prepared using a vacuum manifold and stored in a 2 L bulb to be used as a constant source of BA. Briefly, liquid BA (Sigma-Aldrich, 99%) was placed in a 50 mL bulb and underwent three freeze–pump–thaw cycles. The 50 mL bulb was opened to the vacuum manifold and the 2 L bulb to reach a known BA pressure, which was then diluted in helium (Praxair, 99.999%) to a total pressure of ∼20 kPa.

Initially, the sample was covered using the movable lid, and the temperature of the sample was adjusted to a selected value. The amine was introduced into the cell until a stable signal in the QMS (denoted as *I*_0_) was obtained, and the lid was then lifted to expose the diacid to the gaseous amine. The signal decreased to another steady state (denoted as *I*_r_) due to uptake on the diacid. Note that the vapor pressures of solid glutaric acid and succinic acid are less than 1 × 10^–4^ Pa at 298 K and decrease with decreasing temperature.^32,55^ The low vapor pressures mean that there is negligible reaction in the gas phase.

### 2.3 Uptake Coefficient Calculation

Uptake coefficients (γ) were calculated using eq I,
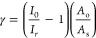
Iwhere *I*_0_ and *I*_*r*_ are corrected by subtracting background signals as described in the Supporting Information and Figure S1. *A*_o_ and *A*_s_ are the areas of the variable orifice and diacid sample, respectively. There was some uptake of BA on the empty sample cup, with an ∼10% decrease in *I*_0_ down to 170 K and much larger decreases below that (Figure S2). This limited the uptake studies to *T* > 175 K. A correction as described in the Supporting Information was applied to *I*_0_ over the temperature range from 177 to 298 K to account for uptake in the absence of the diacid.

### 2.4 Viscosity Measurements

The viscosity measurements for the BA:GA = 1:1 or 2:1 ionic liquids are described in detail in the supporting information (Text S2). Briefly, the synthesized ionic liquids were equilibrated using baths at 298, 273, 238, and 195 K for 10 min, and the viscosities were then measured using the falling sphere viscometer technique.^56^

### 2.5 KM-SUB Modeling Studies

The kinetic multi-layer model of aerosol surface and bulk chemistry (KM-SUB)^51^ was applied to better understand the contributions of different physical and chemical processes to the uptake of BA on GA. KM-SUB treated reversible adsorption to the surface of the acid, partitioning into the bulk and bulk diffusion. Two condensed-phase reactions are considered as glutaric acid is diacid containing two acid groups that can react sequentially with BA:
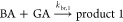
R1
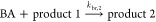
R2where product 1 and product 2 represent the reaction products (i.e., ILs) of BA and GA with mole ratios of 1:1 and 2:1, respectively.

Viscosity was treated as being dependent on the composition of the film at a given depth^57,58^ using eq II,
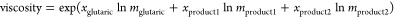
IIwhere *x*_glutaric_, *x*_product1_, and *x*_product2_ are the mole fractions of GA, product 1, and product 2, respectively, at a given depth. Viscosities of pure GA, product 1, and product 2 are denoted as μ_glutaric_, μ_product1_, and μ_product2_, respectively. Viscosities were converted to bulk diffusion coefficients using the Stokes-Einstein equation.

The effective desorption rate coefficient of BA from the surface (*k*_d,eff_) was treated as being dependent on the surface composition, eq III,
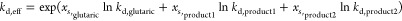
IIIwhere *x*_*s*__,glutaric_, *x*_*s*__,product1_, and *x*_*s*__,product2_ are the mole fractions of GA, product 1, and product 2, respectively, at the surface. *k*_d,glutaric_, *k*_d,product1_, and *k*_d,product2_ are the BA desorption rate coefficients from the surfaces of pure GA, product 1, and product 2, respectively. The effective desorption rate is assumed to be described by an exponential function similar to the viscosity equation (eq II), while we note that further experimental measurements or theoretical calculations would be required to determine accuracy of this treatment.

Temperature-dependent values of parameters used in the model were plotted for reaction rate coefficients (Figure S3a), desorption rate coefficients for BA from different surfaces (Figure S3b), viscosity (Figure S3c), and are summarized in Table S1. Temperature-dependent parameters were also constrained at room temperature by fitting to measurements with changing sample mass and BA initial concentrations ([BA]_0_). The temperature dependence of the reaction rate coefficients and the desorption rates were treated using Arrhenius equations. The temperature-dependencies of the viscosities were treated using the modified Vogel–Tammann–Fulcher equation.^59^ Unknown parameters were determined by using the Monte Carlo Genetic Algorithm (MCGA).^60^ The unknown parameters at room temperature that were determined by the MCGA by fitting to the BA concentration dependence data were *k*_br,1,_*k*_br,2,_*k*_d,glutaric_, *k*_d,product1_, *k*_d,product2_, μ_product1_, and μ_product2_. The MCGA was subsequently run to fit to the temperature-dependence data and to determine activation energies of the reaction rate coefficients and desorption rate coefficients. Parameters which were used to determine the temperature dependence of the viscosities were fixed to match the value determined at 298 K and the measured viscosity at the lowest temperature.

## 3 Results and Discussion

### 3.1 Reactive Uptake Profiles at Room Temperature

**Figure 2:**
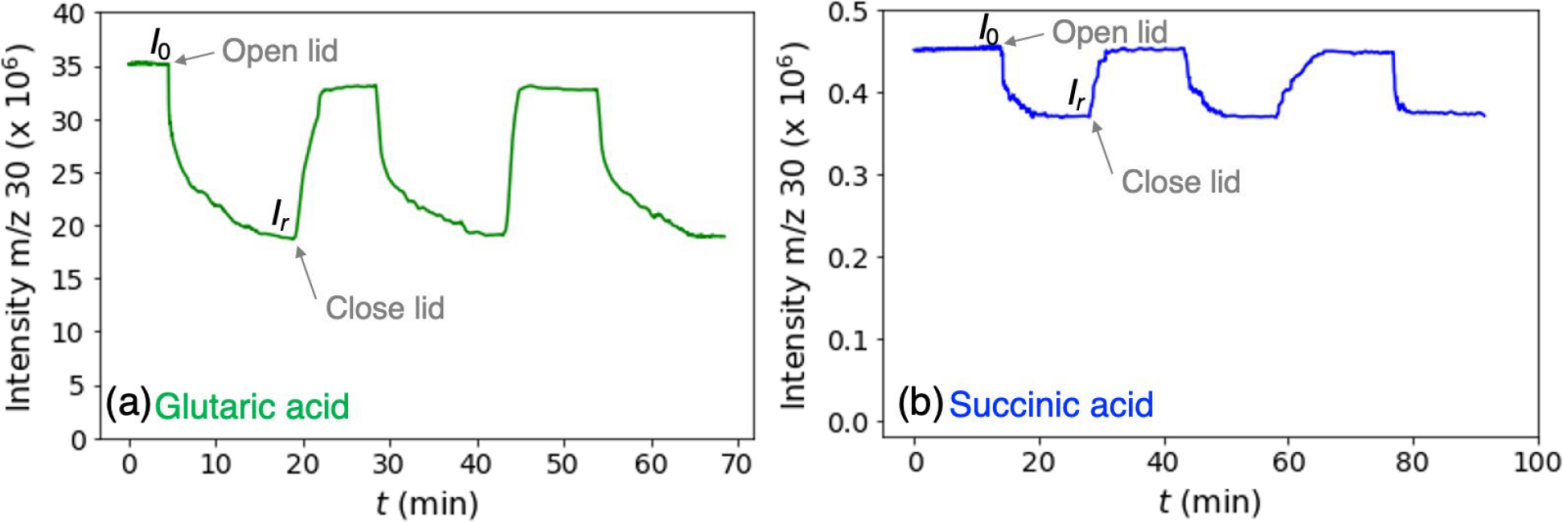
Temporal profiles of the BA signal for three consecutive uptake measurements on (a) 0.0310 g glutaric acid and (b) 0.1540 g succinic acid at 298 K. The initial concentration of BA is 4.0 × 10^11^ molecules cm^–3^, and the change in its concentration is tracked by the intensity of the fragment at *m*/*z* = 30. The effective areas of the variable orifices are 0.33 cm^2^ for glutaric acid and 0.011 cm^2^ for succinic acid.

Figure 2 shows time profiles for uptake of BA on GA and SA at 298 K. Initially, the signal at *m/z* 30 is in a steady state (*I*_0_) with the sample covered. When the sample is exposed to BA by opening the lid, the signal decreases due to the uptake on the diacid and reaches a new steady state (*I*_r_). It takes longer to reach the new steady state in the case of GA compared to SA. Amines are notoriously “sticky” gases that tend to reversibly adsorb on surfaces, including the walls of the chamber. The higher uptake on GA results in a larger decrease in the BA concentration in the cell when the sample lid is opened. This decrease in BA concentration perturbs the equilibrium between BA in the gas phase and on the chamber walls, causing desorption of BA from the walls. This desorption is relatively slow, resulting in longer times to reach steady state for GA compared to that of SA where the change in the BA concentration due to uptake on the diacid is smaller. For both diacids, the time profiles are consistent amongst three consecutive measurements (Figure 2).

Uptake coefficients (γ) can be obtained from the decrease in the BA signals on opening the lid to expose the sample, combined with the areas of the sample and orifice connected to the mass spectrometer (eq I). These are summarized in Table 1 for GA and Table 2 for SA. The uptake coefficients of BA at 298 K are 0.11 ± 0.04 on GA and (1.9 ± 0.5) × 10^–4^ on SA. These are consistent with previous measurements at room temperature by Fairhurst et al.^43^ at similar concentrations but with a Knudson cell of a different design that reported an uptake coefficient of 0.10 ± 0.024 on GA and (1.7 ± 0.6) × 10^–4^ on SA. The value reported by Gao et al.^50^ for uptake of BA on SA at 295 K, (3.8 ± 0.1) × 10^–4^, is larger than that reported here due to the use of BA concentrations that are about an order of magnitude higher than in the present study, but consistent with earlier work at these higher concentrations where the uptake of BA at (4.9 ± 0.5) × 10^12^ molecules cm^–3^ on SA was (3.3 ± 0.9) × 10^–4^.^43^

**Table 1:** Uptake Coefficients (γ ± 2σ) for BA on Glutaric Acid (GA) as a Function of Temperature^a^

*T* (K)	GA mass (g)	GA surface area (cm^2^)	γ	averaged γ ± 2σ^b^
298	0.0175–0.0943	1.1–6.2		0.11 ± 0.04^c^
288	0.0360	2.4	0.19	0.17 ± 0.06
	0.0318	2.1	0.14	
278	0.0311	2.0	0.17	0.19 ± 0.04
	0.0356	2.3	0.21	
268	0.0311	2.0	0.18	0.20 ± 0.04
	0.0323	2.1	0.20	
	0.0324	2.1	0.24	
258	0.0283	1.9	0.20	0.22 ± 0.03
	0.0289	1.9	0.22	
	0.0323	2.1	0.23	
248	0.0296	1.9	0.25	0.25 ± 0.04
	0.0296	1.9	0.23	
	0.0323	2.1	0.27	
243	0.0318	2.1	0.18	0.20 ± 0.05
	0.0357	2.3	0.22	
238	0.0251	1.7	0.067	0.076 ± 0.028
	0.0357	2.3	0.092	
	0.0369	2.4	0.068	
228	0.0250	1.6	0.048	0.053 ± 0.014
	0.0269	1.8	0.058	
218	0.0268	1.8	0.053	0.049 ± 0.012
	0.0294	1.9	0.045	
208	0.0269	1.8	0.055	0.046 ± 0.028
	0.0282	1.9	0.036	
198	0.0290	1.9	0.046	0.040 ± 0.017
	0.0360	2.4	0.034	
188	0.0298	2.0	0.051	0.038 ± 0.038
	0.0356	2.3	0.024	
177	0.0289	1.9	0.034	0.030 ± 0.010
	0.0411	2.7	0.027	

a Orifice area is 0.33 cm^2^, and [BA]_0_ is 4.0 × 10^11^ molecules cm^–3^.

b Uncertainties represent 2σ statistical errors. Overall error includes possible systematic errors estimated to be ±60% (from error propagation based on eq I) due to uncertainties associated with sample surface areas of the polydisperse powders (± 32%) and the correction to *I*_0_ and *I*_r_ from the BA background (resulting in a relative error for (*I*_0_/*I*_r_ −1) up to ±51%).

c Averaged from 19 points summarized in Table S2.

**Table 2:** Uptake Coefficients (γ ± 2σ) for BA on Succinic Acid (SA) as a Function of Temperature^a^

*T* (K)	SA mass (g)	SA surface area (cm^2^)	γ	averaged γ ± 2σ^b^
298	0.0500–0.3025	3.6–22		(1.9 ± 0.5) × 10^–4^^c^
288	0.0708	5.1	3.0 × 10^–4^	(4.3 ± 4.0) × 10^–4^
	0.0973	7.0	5.7 × 10^–4^	
278	0.0967	7.0	1.3 × 10^–3^	(1.1 ± 0.6) × 10^–3^
	0.1998	14	8.5 × 10^–4^	
268	0.1407	10	1.3 × 10^–3^	(1.5 ± 0.4) × 10^–3^
	0.1719	12	1.6 × 10^–3^	
258	0.0769	5.5	2.2 × 10^–3^	(2.1 ± 0.2) × 10^–3^
	0.1028	7.4	2.1 × 10^–3^	
248	0.0583	4.2	2.9 × 10^–3^	(2.9 ± 0.1) × 10^–3^
	0.0787	5.7	3.0 × 10^–3^	
238	0.1027	7.4	5.5 × 10^–3^	(5.7 ± 0.5) × 10^–3^
	0.0969	7.0	5.9 × 10^–3^	
228	0.0777	5.6	9.6 × 10^–3^	(1.2 ± 0.8) × 10^–2^
	0.0913	6.6	1.5 × 10^–2^	
218	0.0715	5.2	2.8 × 10^–2^	(2.8 ± 0.1) × 10^–2^
	0.0777	5.6	2.9 × 10^–2^	
208	0.0622	4.5	4.8 × 10^–2^	(4.4 ± 1.0) × 10^–2^
	0.0791	5.7	4.0 × 10^–2^	
198	0.0767	5.5	8.2 × 10^–2^	(7.5 ± 2.2) × 10^–2^
	0.0770	5.5	6.7 × 10^–2^	
188	0.0777	5.6	9.4 × 10^–2^	(1.1 ± 0.3) × 10^–1^
	0.0785	5.7	1.2 × 10^–1^	
177	0.0777	5.6	1.2 × 10^–1^	(1.4 ± 0.5) × 10^–1^
	0.0877	6.3	1.6 × 10^–1^	

a Orifice areas are 0.011 cm^2^ for temperatures ranging from 298 to 238 K and 0.33 cm^2^ for 228 to 177 K. [BA]_0_ is 4.0 × 10^11^ molecules cm^–3^.

b Uncertainties represent 2σ statistical errors. Overall error includes possible systematic errors estimated to be ±60% due to uncertainties associated with sample surface areas of the polydisperse powders and the correction to *I*_0_ and *I*_r_ from the BA background.

c Averaged from 17 points summarized in Table S3.

### 3.2 Dependence of Uptake Coefficients on Sample Mass and BA Initial Concentration ([BA]_0_)

**Figure 3:**
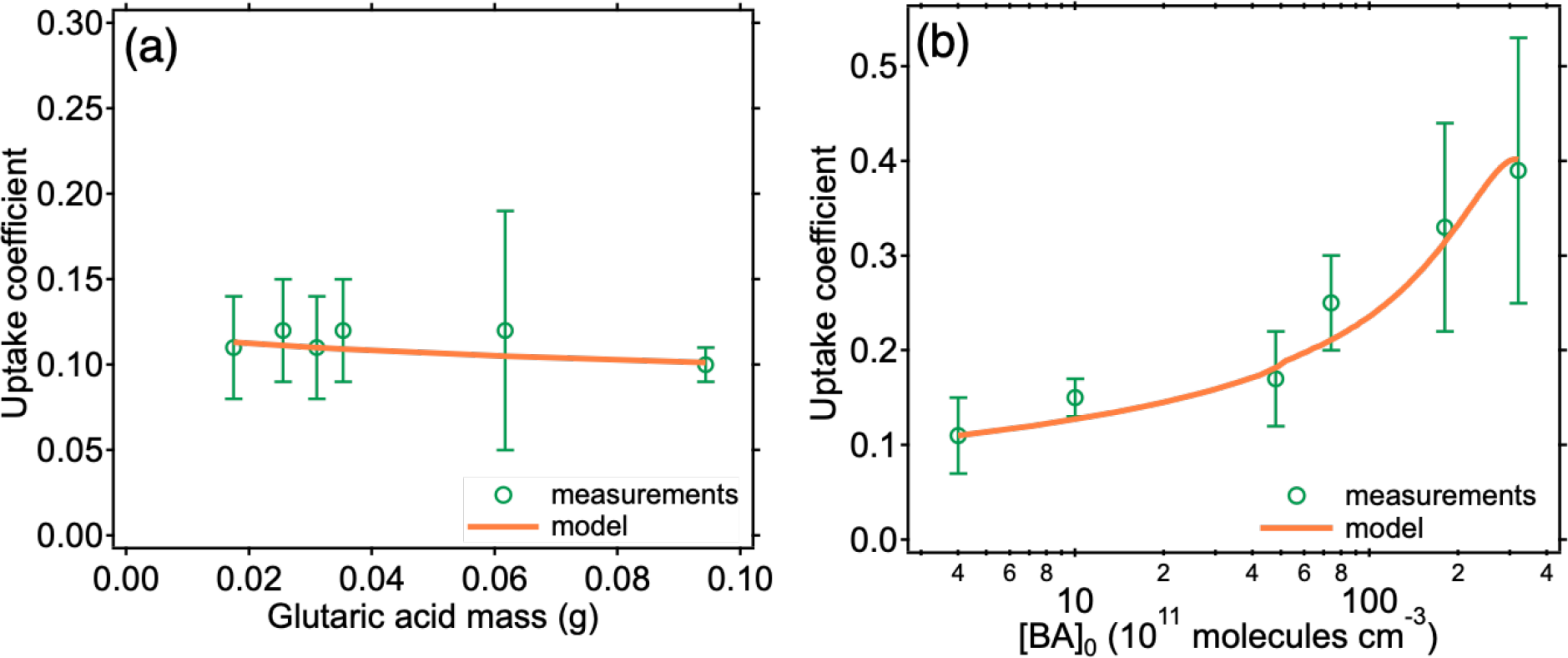
Dependence of measured (green circles) and KM-SUB modeled (orange lines) uptake coefficients of BA on glutaric acid at 298 K on (a) sample mass from 0.0175 to 0.0943 g with a constant [BA]^0^ of 4 × 10^11^ molecules cm^–3^ and (b) BA initial concentration from 4 × 10^11^ to 3.2 × 10^13^ molecules cm^–3^ at a glutaric acid mass of 0.0175–0.0943 g. The error bars denote two standard deviations of at least two (*n* ≥ 2) repeated measurements.

The sample mass of GA was varied from 0.0175 to 0.0943 g to examine its impact on the uptake coefficient (Figure 3a and Table S2). Similarly, uptake coefficients of BA on SA with the mass of 0.0500 g to 0.3025 g were measured (Table S3). The measured uptake coefficients show little dependence on sample mass for both GA and SA, consistent with a previous study and as expected for less than a single layer of particles in the Knudsen cell.^43,54^ This lack of mass dependence is reproduced well by the kinetic multi-layer model KM-SUB (Figure 3a).^51^

To probe the dependence of the uptake coefficients on BA initial concentration, [BA]_0_ was varied from 4.0 × 10^11^ molecules cm^–3^ to 3.2 × 10^13^ molecules cm^–3^ in the GA experiments (Figure 3b and Table S4). There is an increasing trend of uptake coefficients from 0.11 ± 0.04 to 0.39 ± 0.14 with increasing [BA]_0_. KM-SUB model results suggest that this increasing trend is caused by more products being formed near the surface, leading to significant decreases in viscosity (Figure S4) and BA desorption rate coefficients (Figure S5) compared to those of solid GA.

### 3.3 Dependence of Uptake Coefficients on Temperature

**Figure 4:**
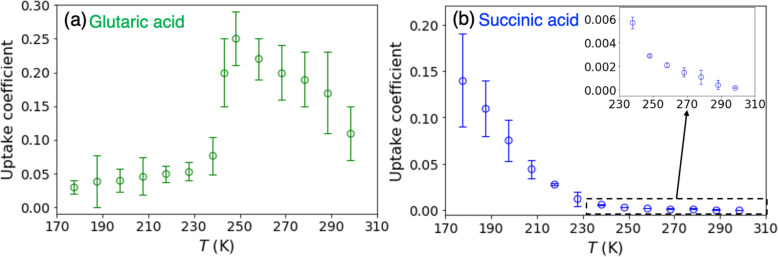
Temperature-dependence of measured uptake coefficients of BA on (a) glutaric acid and (b) succinic acid from 177 to 298 K. The initial concentration of BA is 4 × 10^11^ molecules cm^–3^. The error bars denote two standard deviations of at least two (*n* ≥ 2) repeated measurements.

To compare the temperature dependence of reactive uptake processes with and without the formation of IL layers, the uptake coefficients of BA on GA and SA were measured as a function of temperature from 298 to 177 K (Figure 4 and Tables 1 and 2). Overall, the uptake coefficients of BA on GA are larger than those on SA at *T* > 208 K, consistent with previous studies showing that the reactive uptake of amine/ammonia on odd carbon diacids is faster than that on even carbon diacids due to the formation of IL layers on the former.^26,43^

The trends in the temperature dependence of the uptake coefficients for the two diacids are distinct. The measured uptake coefficients for GA increase from 298 to 248 K and then fall with a further decrease in temperature (Figure 4a, Table 1), while for SA there is a continuous increase with decreasing temperature (Figure 4b, Table 2).

Reactive uptake of BA on the diacids is a complex, multi-step process involving adsorption at the surface, desorption back into the gas phase, and reaction between the acid and base starting at the surface, and through diffusive processes, working down into the solid. Thus, there is an interplay between non-reactive interactions, reaction kinetics, and molecular diffusion that change with time and layer-by-layer in the diacid particles.^51^ In the case of GA, reaction leads to the formation of an IL layer^43^ into which the BA is taken up and the underlying GA dissolves. This results in fast uptake at room temperature.^43^ As the temperature decreases, these processes play different roles in changing the uptake coefficients. One of the important factors contributing to the temperature dependence of the uptake coefficients on GA is the change in the viscosity of the IL layer as a function of temperature. The measured viscosities for the mixtures of BA and GA in both 1:1 and 2:1 mole ratios show a dramatic increase with decreasing temperature from 298 to 195 K as described in Figure S3c.

**Figure 5:**
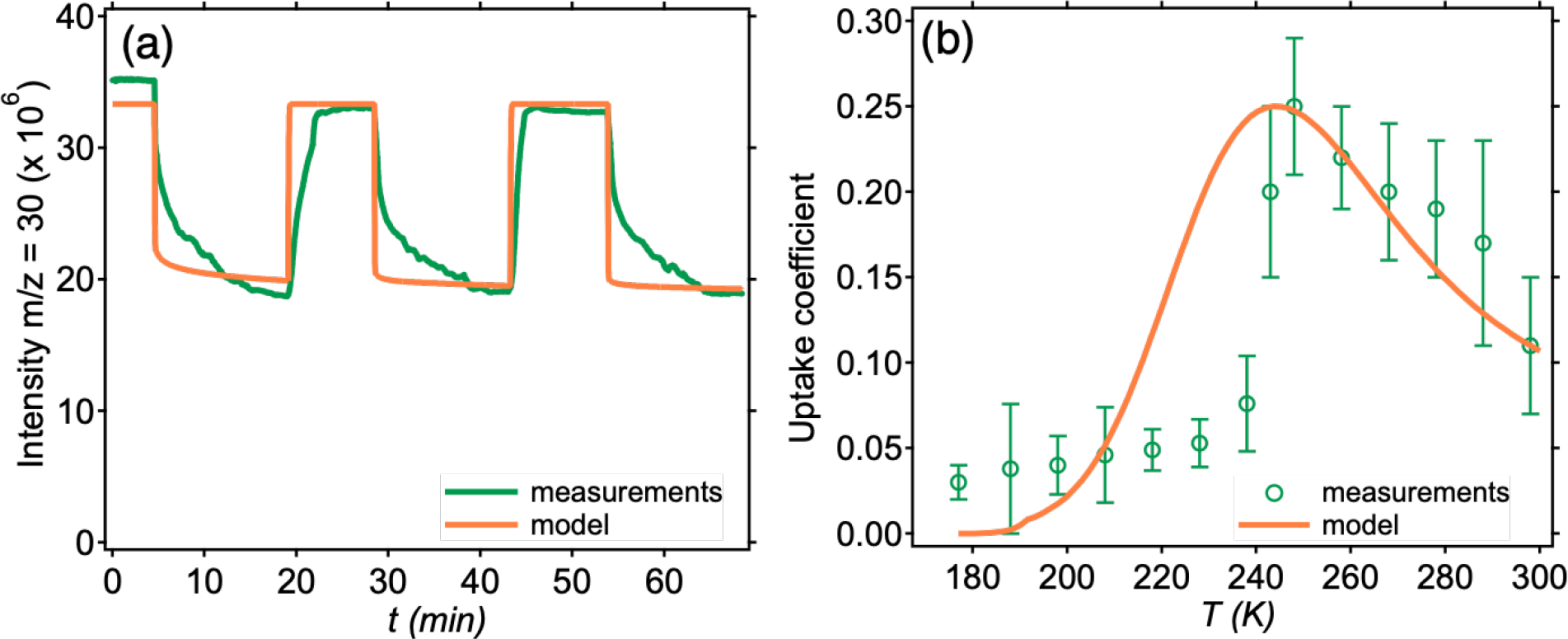
Comparison of experimental measurements and KM-SUB modeling results for the uptake of BA on glutaric acid. (a) Measured (green line) and modeled (orange line) temporal profiles of BA signal during uptake measurements at 298 K. (b) Measurements (green circles) and modeling (orange line) of the BA uptake coefficients as a function of the sample temperature.

To elucidate the relative importance of the different physical and chemical processes contributing to BA uptake as a function of temperature, KM-SUB was applied.^51^Figure 5a shows the experimental temporal uptake profiles for BA on GA at room temperature and the predictions from KM-SUB. The model reproduces the overall trend and steady states in the signal reasonably well but predicts a faster initial decay in the signal upon exposure than observations. This is likely due to slow desorption of BA from the chamber walls in the experiment which is difficult to include in the model. Figure 5b shows the comparison between the measured uptake coefficients of BA on GA as a function of temperature and the predictions from KM-SUB. Overall, the model reproduces the unique trend of uptake coefficients with temperature over this wide range of temperatures. The increase in uptake coefficients from 298 to 248 K is dominated by a decrease in the desorption rate coefficients of BA from the surfaces, despite the slower reaction kinetics (Figure S3). The model also predicts that the decrease in uptake coefficients with decreasing temperature below 248 K is due to a decrease in the reaction rate coefficients (both *k*_br,1_ and *k*_br,2_) and a significant increase in viscosity (Figure S3). Thus, a competition between these processes leads to the unique temperature-dependence.

The rapid initial drop in the uptake coefficient below 248 K followed by a slow decline with temperature is not fully reproduced by the model (Figure 5b). It is possible that some of the assumptions built into the model (e.g., Stokes-Einstein relationship) may not be applicable in ILs at these low temperatures. There may also be a missing and untreated process in the model such as freezing of BA on the surface at very low temperatures.

For SA, there is a continuous increase in the uptake coefficients as the temperature decreases (Figure 4b, Table 2). Reactions of the even carbon diacids with amines do not form measurable ILs, so that the mechanism involves formation of solid salts at the surface followed by diffusion into the solid and reaction in the underlying layers. The high viscosity of the solid diacid and its aminium salts compared to the IL layers formed on the odd carbon diacids slows diffusion into the underlying diacid structure so that adsorption and desorption become relatively more important compared to reaction kinetics. The continuous increase in uptake of BA on SA as the temperature decreases from 298 K is consistent with a slower rate of desorption of BA from the surface at lower temperatures, similar to GA in the 298–248 K range. However, KM-SUB could not readily capture the trend in uptake down to 177 K. KM-SUB model sensitivity tests indicated that high uptake coefficients measured at low temperatures could not be explained by reactions between BA and succinic acid. This is because diffusion limitations of both succinic acid and BA would be too great, causing succinic acid to become rapidly depleted near the surface and limiting the concentration of BA penetrating into the solid bulk. The high uptake coefficients may be due to multi-layer adsorption of BA on the surface at the lowest temperatures (the freezing point of BA is 224 K^53^).

**Figure 6:**
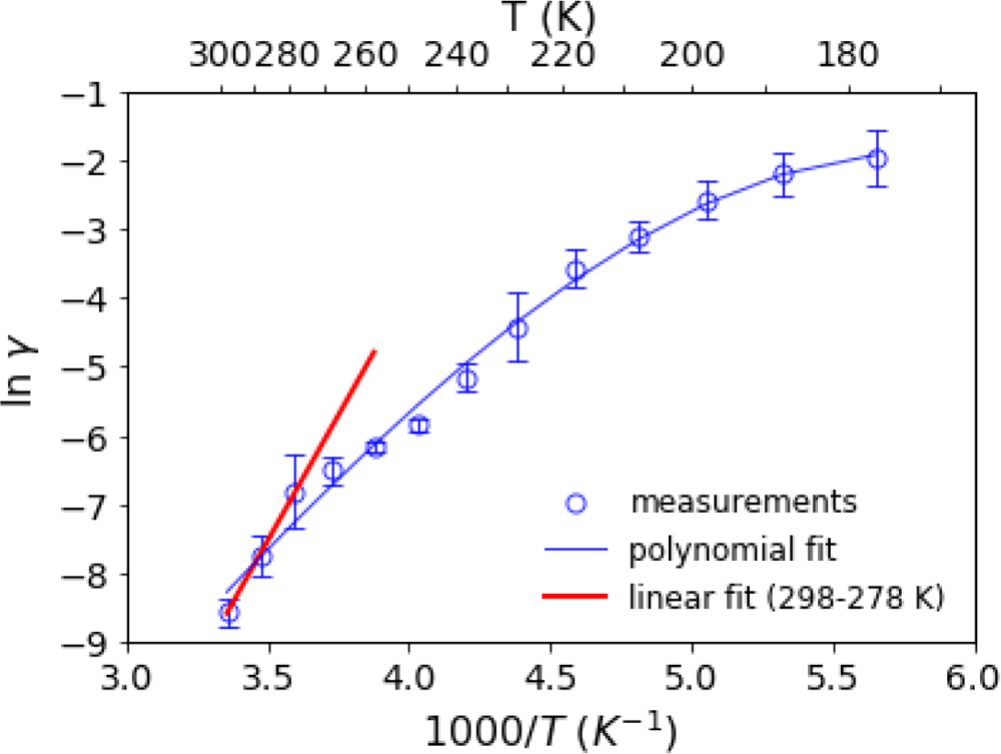
Measured (blue circles) values of ln γ vs 1/*T* for the uptake of BA on succinic acid at temperatures ranging from 298 to 177 K. The blue line is a third-order polynomial fit to all data as a guide to the eye. The red line is the linear regression from 298 to 278 K: ln γ = (7280 K)/*T* – 33.0 (*R*^2^ = 0.9996). The error bars denote two standard deviations of at least two (*n* ≥ 2) repeated measurements.

Uptake coefficients can be expressed as rate constants (*k*, cm^–2^ s^–1^) via γ = 4*V*/*u*_av_ × *k*, where *u*_av_ is the thermal velocity of BA molecules and *V* is the volume of the cell.^1^ Assuming the rate constant and, hence, uptake coefficient follow an Arrhenius dependence on temperature, an effective activation energy can be obtained from a plot of ln γ versus 1/*T*. Figure 6 shows such a plot for SA over the entire temperature range, with a polynomial fit to the data. To compare to the previous study by Gao et al.^50^ that reported an activation energy of −71.9 kJ mol^–1^ over the temperature range from 295–263 K, linear regression was applied to the data in Figure 6 over a similar temperature range, from 298–278 K, giving an effective activation energy of (−61 ± 20) kJ mol^–1^. In the previous study by Gao et al., the formation of the carboxylate group in the products was followed with time giving an activation energy that reflects the net reaction. The present study measured the uptake of BA, therefore the activation energy reflects both chemical and physical processes (e.g., adsorption/desorption of BA, diffusion). Nevertheless, our value of (−61 ± 20) kJ mol^–1^ over a similar temperature range is consistent with their result.

## 4 Summary and Conclusions

With our newly designed temperature-controlled Knudsen cell system, the reactive uptake coefficients for BA on GA and SA over a wide temperature range (177–298 K) that covers most tropospheric conditions were measured for the first time. The temperature dependences of uptake coefficients are distinct for the two solid diacids in the same homologous series. While the uptake coefficients on SA increase monotonically from 298 to 177 K, those on GA increase from 298 to 248 K but then decrease from 248 to 177 K. This difference is consistent with the previously proposed mechanism for uptake of BA on the two diacids.^43^ While the reactive uptake on SA is by a surface reaction mechanism to form solid aminium carboxylate salts with desorption of BA being a controlling factor, that on GA forms ILs and is controlled by a set of processes including the desorption of BA, dissolving of GA and BA in the IL layer, diffusion in the IL layer, and acid–base reactions. This is supported by quantitative modeling using the KM-SUB which parameterizes the temperature dependences of the key steps involved in this reactive uptake.

These combined experimental and modeling studies provide a quantitative understanding and a unique predictive capability for such multi-step, complicated uptake processes. They provide guidance on accurately quantifying the reactive uptake processes involving phase changes at various temperatures in atmospheric models.^48^ Additionally, these two distinct reactive uptake systems provide a benchmark for understanding how the dominant steps of multiphase processes change with temperature and the particle phase state. Further understanding and parameterization of the thermodynamic and kinetic components involved in the uptake processes for other systems will be important for accurately predicting the impacts of organic particles on human health and welfare.

## Supporting Information Available

Correction of *I*_0_ and *I*_r_ for background *n*-butylamine (BA) signals and uptake on empty sample cup at low temperatures (Text S1, Figures S1, S2); preparation of ionic liquids (ILs) and viscosity measurements (Text S2); KM-SUB predicted temperature dependence of the reaction rate coefficients, the desorption rate coefficients, and viscosities (lines) and the viscosities (circles) from measurements (Figure S3); KM-SUB predicted viscosity as a function of time and depth for the GA reaction at two different BA concentrations (Figure S4) and predicted effective desorption rate coefficients as a function of time and BA concentration (Figure S5); parameters used in the KM-SUB model (Table S1); uptake coefficients for BA on GA and SA as a function of sample mass at 298 K (Tables S2 and S3); and uptake coefficients for BA on GA as a function of BA initial concentration (Table S4) (PDF)
Visual recordings of the motion of the IL materials (ZIP)
