# Supplementary material for: Distinct Temperature Trends in the Uptake of Gaseous n-Butylamine on Two Solid Diacids
Source: ACS EST Air. 2023 Nov 29;1(1):52–61. doi: 10.1021/acsestair.3c00032 (PMC10798143; doi:10.1021/acsestair.3c00032)
Supplement: Supplementary file 1 — ea3c00032_si_001.pdf [file ea3c00032_si_001.pdf]

Supporting Information for:

Distinct Temperature Trends in the Uptake of Gaseous *n*-Butylamine on Two Solid Diacids

Yixin Li,[a] Pascale S. J. Lakey,[a] Michael J. Ezell,[a] Kristen N. Johnson,[a] Manabu Shiraiwa,[a]* and Barbara J. Finlayson-Pitts[a]**

[a] Department of Chemistry, University of California, Irvine, Irvine, CA 92697-2025

*To whom correspondence on multiphase kinetics modeling should be addressed: m.shiraiwa@uci.edu

** To whom correspondence on experiments should be addressed: bjfinlay@uci.edu

Text S1:

Correction of I_0 and I_r for background *n*-butylamine (BA) signals and uptake on empty sample cup at low temperatures. Amines, including BA, adsorb readily on surfaces and desorb slowly on pumping. To correct I_0 and I_r for background BA signals, the flow of BA was shut off after a steady state was reached on exposure of the diacid to the BA and the decay was followed with time. The residual signal after 5 min was used as the background signal, I_b, to subtract from I_r and the subsequent I_0 when the sample lid was closed off and the BA flow restored (Figure S1). This was done for each value of I_r and the subsequent I_0.

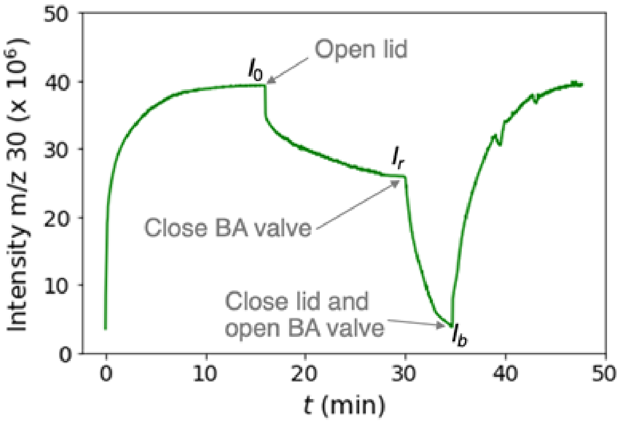

Figure S1. Correction of I_0 and I_r for the background from BA. Temperature was 298 K, the sample mass of glutaric acid was 0.0255 g and BA initial concentration was 4×10^{11} molec cm^{-3}. Orifice area was 0.33 cm^2.

The system was designed to cool the bottom of the sample cup and hence control the temperature of the sample. The walls of the sample cup that connected it to the main chamber were made of thin stainless steel whose thermal conductivity was small. However, over the course of a number of hours required for the experiments, some cooling of the walls occurred, resulting in uptake of BA at temperatures below 298 K. Figure S2 shows the decrease in BA signal when the empty

sample cup was cooled. A small decrease (~10%) was observed down to ~170 K but then there was a dramatic drop at lower temperatures. As a result, measurements were restricted to T > 175 K, and corrections were applied to I_0 for this uptake.

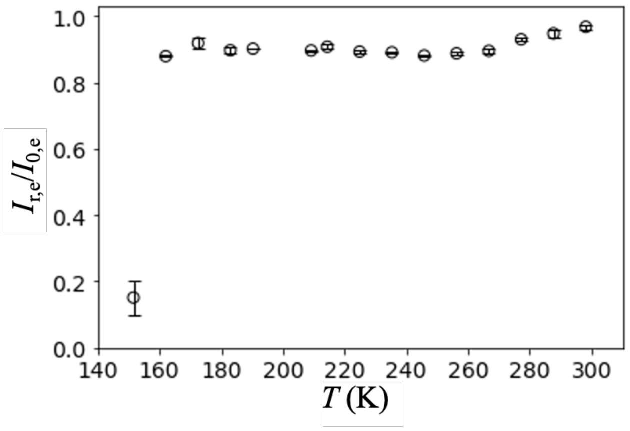

Figure S2. Ratio of the BA signal intensity on opening to the empty sample cup ($I_{r,e}$) to that when the empty sample cup was closed off ($I_{0,e}$) as a function of temperature, showing ~10% loss down to 175 K. The error bars denote two standard deviations of at least two ($n \geq 2$) repeated measurements.

Text S2:

Preparation of ionic liquids (ILs) and viscosity measurements. Glutaric acid (GA, 2.5 g) was dissolved in 300 mL nanopure water (18.2 MΩ cm) and then mixed with either 1.87 or 3.74 mL of liquid BA to give salts with 1:1 or 2:1 mole ratios of BA to GA. The water was removed by evaporation using a rotovap (Wheaton, SPIN-VAP) at 20 kPa and 70~75 ºC. The resulting ionic liquids were pumped under vacuum of < 1 Pa for 8 hr to remove the remaining water.

Viscosity was measured using the falling sphere viscometer technique.[1] The dried liquid was placed in a 5 mL graduated cylinder and a metal sphere of known density (ρ_S = 4.2 × 10^3 kg

m^{-3}) and radius (r = 0.51 mm) was allowed to fall through the liquid. The viscosity of the liquid (μ) is given by Eq S1,[1]

$$\mu = \frac{2gr^2(\rho_s - \rho_l)}{9v} \quad \text{(Eq S1)}$$

where g is the gravitational constant, ρ_l is the density of the ionic liquid (ρ_l =1.1 × 10^3 kg m^{-3}),[2] and v is the velocity of the sphere falling through the liquid. Cooling baths were prepared in order to measure viscosities at selected temperatures. These included an ice/water bath for 273 K, an ice/$CaCl_2$ water solution for 238 K, dry ice/acetone for 195 K, and liquid nitrogen for 77 K. The graduated cylinder containing the liquids and the sphere were equilibrated with each cooling bath for 10 min before each measurement. The measurements were carried out with the liquid submerged in the bath.

The viscosities were measured at several temperatures from 195 K to 298 K; visual recordings of the motion of the material were also made (see Web Enhanced Objects). Both the 1:1 and 2:1 BA:GA salts are liquid at room temperature and become more and more viscous with decreasing temperatures. At temperature below around 238 K, both salts become semi-solids,[3] and in liquid nitrogen (77 K), both salts form solid with cracks due to rapid cooling. This increase in the viscosities plays a significant role in driving the decrease in uptake coefficients at decreasing temperatures below 248 K in the GA case (Figure 4a).

The KM-SUB model was not able to match the experimental observations for the uptake on GA using the measured temperature-dependent viscosities (Figure S3c). This discrepancy is possibly due to IL not obeying the Stokes-Einstein equation or proton hopping where protons instead of GA molecules diffuse in the layer of IL by hopping between neighboring glutarate ions.[4] Another possible reason is that the micro-viscosity of IL formed on particles could be significantly smaller than the measured bulk viscosity.[5]

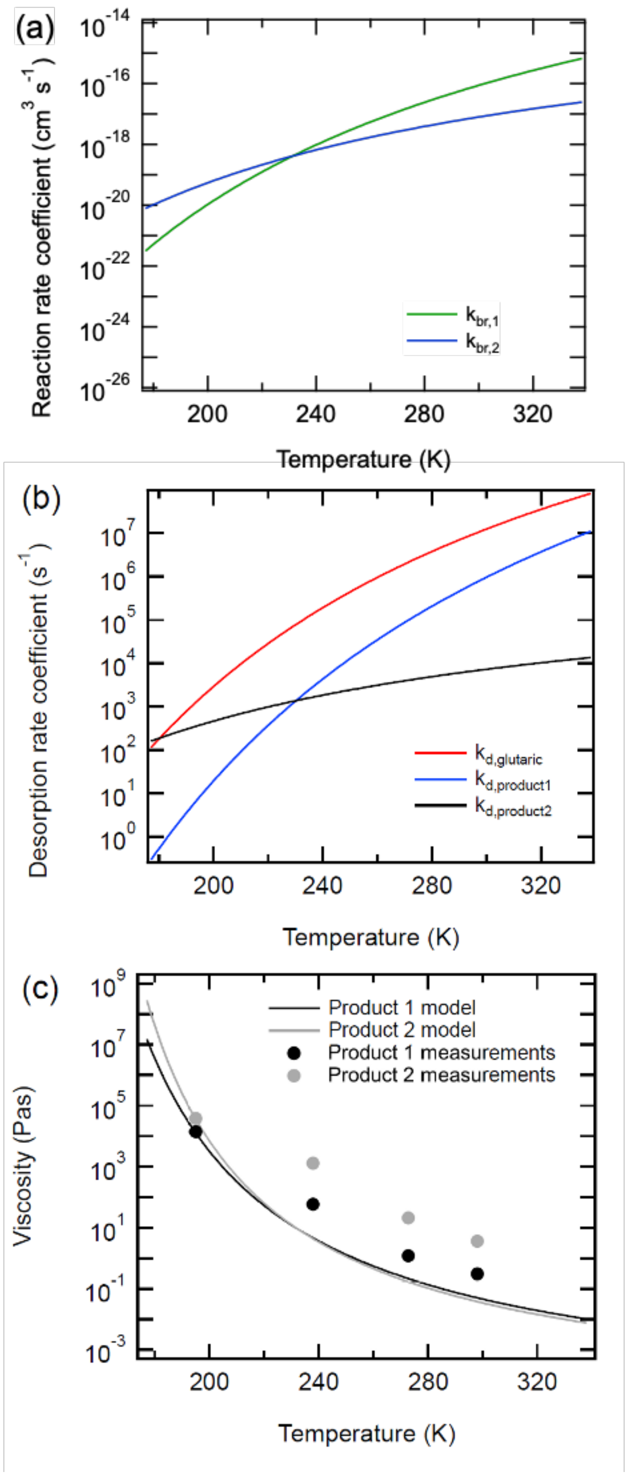

Figure S3. KM-SUB predicted temperature dependence (lines) of (a) reaction rate coefficients, (b) desorption rate coefficients and (c) viscosities. Circles in (c) are the measured values.

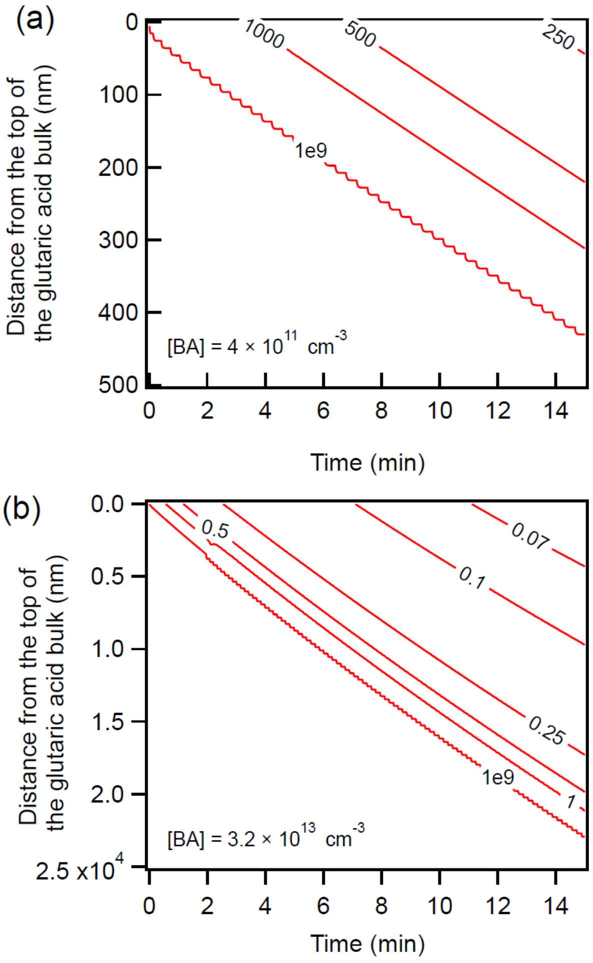

Figure S4: Viscosity as a function of time and depth at 298 K predicted by the model for the (a) lowest and (b) highest BA concentrations at which experiments were conducted. Note that step-like changes are an artifact of the model and are caused by having multi-layers in KM-SUB. Contour lines are in units of Pa s.

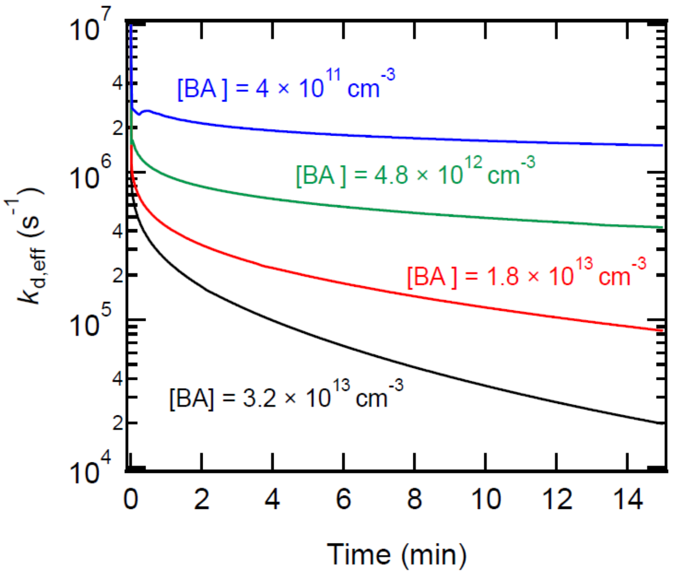

Figure S5: The effective desorption rate coefficients for BA from the surface as a function of time outputted from the model for different BA concentrations at which experiments were performed.

Table S1. Parameters used in the KM-SUB model. Note that gas constant R = 0.0083 kJ mol^{-1} K^{-1}. T is in units of Kelvin.

Parameter	Description	Value	Notes
$[BA]_g$	BA gas-phase concentration	4 × 10^{11} cm^{-3} unless otherwise stated	Experimental value
T	Temperature	298 K unless otherwise stated	Experimental value
S	Glutaric acid surface area	2.03 cm^2 unless otherwise stated	Experimental value
M_{GA}	Glutaric acid mass	0.031 g unless otherwise stated	Experimental value
t	Reaction time	15 minutes	Experimental value
$A_{orifice}$	Orifice surface area	0.33 cm^2	Experimental value
α_s	Surface mass accommodation of BA onto an adsorbate-free surface	1	
$k_{br,1}$ (cm^3 s^{-1})	Reaction rate coefficient of glutaric acid with BA	5.99×10^{-9}×exp(-44.9/(RT))	Note that surface rate coefficients (k_{sr}) were treated as follows: $k_{sr,1}$=$k_{br,1}$/$\delta_{glutaric}$ (cm^2 s^{-1}) $k_{sr,2}$=$k_{br,2}$/$\delta_{product1}$ (cm^2 s^{-1}) where δ is the diameter of a molecule. $\delta_{glutaric}$ = 0.55 nm $\delta_{product1}$ = 0.70 nm
$k_{br,2}$ (cm^3 s^{-1})	Reaction rate coefficient of Product 1 with BA	1.70×10^{-13}×exp(-24.8/(RT))	
K_{BA} (mol cm^{-3} atm^{-1})	Partitioning coefficient of BA	1 × 10^{-3}	Insensitive as the reaction mainly occurs at the surface.
$k_{d,glutaric}$ (s^{-1})	Desorption rate coefficient of BA from the glutaric acid surface	2.29×10^{14}×exp(-41.6/(RT))	Pre-exponential factors are within the typical range of values for glutaric acid and product 1.* The pre-exponential factor is a bit low for product 2 but this may be a consequence of the model being simplified and missing processes in the model.
$k_{d,product1}$ (s^{-1})	Desorption rate coefficient of BA from the product 1 surface	2.38×10^{15}×exp(-53.8/(RT))	
$k_{d,product2}$ (s^{-1})	Desorption rate coefficient of BA from the product 2 surface	1.80×10^6×exp(-13.69/(RT))	
$\mu_{glutaric}$ (Pa s)	Viscosity of glutaric acid	1 × 10^9	Consistent with a solid.
$\mu_{product1}$ (Pa s)	Viscosity of product 1	η = 1×10^{-5}×exp((T_oD)/(T-T_o)) T_o = 124 K D = 11.99	Fixed values were used for glutaric acid to match the viscosities required to fit the 298 K data and to match the measured viscosities at the lowest temperature.
$\mu_{product2}$ (Pa s)	Viscosity of product 2	η = 1×10^{-5}×exp((T_oD)/(T-T_o)) T_o = 133 K D = 10.25	

* M. Shiraiwa, R. M. Garland and U. Pöschl, Kinetic double-layer model of aerosol surface chemistry and gas-particle interactions (K2-SURF): Degradation of polycyclic aromatic hydrocarbons exposed to O_3, NO_2, H_2O, OH and NO_3, *Atmos. Chem. Phys.*, 2009, **9**, 9571-9586.

Table S2. Uptake coefficients ($\gamma \pm 2\sigma$) for BA on glutaric acid (GA) as a function of sample mass at 298 K.[a]

GA mass (g)	GA Surface area (cm^2)	γ	Averaged $\gamma \pm 2\sigma$ [b]
0.0175	1.1	0.10	0.11 ± 0.03
		0.11	
		0.13	
0.0255	1.7	0.13	0.12 ± 0.03
		0.11	
		0.10	
0.0310	2.0	0.11	0.11 ± 0.03
		0.10	
		0.10	
		0.14 [c]	
0.0353	2.3	0.10	0.12 ± 0.03
		0.11	
		0.13	
0.0617	4.0	0.081	0.12 ± 0.07
		0.12	
		0.15	
0.0943	6.2	0.092	0.10 ± 0.01
		0.10	
		0.10	

[a] Orifice area is 0.33 cm^2 and $[BA]_0$ is 4.0×10^{11} molec cm^{-3}.

[b] Uncertainties represent 2σ statistical errors. Overall error includes possible systematic errors estimated to be ± 60% due to uncertainties associated with sample surface areas of the polydisperse powders and the correction to I_0 and I_r from the BA background.

[c] GA mass is 0.0318 g.

Table S3. Uptake coefficients ($\gamma \pm 2\sigma$) for BA on succinic acid (SA) as a function of sample mass at 298 K.[a]

SA mass (g)	SA Surface area (cm^2)	γ	Averaged $\gamma \pm 2\sigma$ [b]
0.0500	3.6	2.0×10^{-4}	$(1.9 \pm 0.2) \times 10^{-4}$
		1.9×10^{-4}	
		1.8×10^{-4}	
0.1030	7.4	2.2×10^{-4}	$(2.0 \pm 0.5) \times 10^{-4}$
		2.2×10^{-4}	
		1.8×10^{-4}	
0.1540	11	1.7×10^{-4}	$(2.0 \pm 1.0) \times 10^{-4}$
		1.7×10^{-4}	
		2.6×10^{-4}	
0.1800	13	1.9×10^{-4}	$(1.9 \pm 0.2) \times 10^{-4}$
		2.0×10^{-4}	
		1.8×10^{-4}	
0.3025	22	2.1×10^{-4}	$(1.9 \pm 0.4) \times 10^{-4}$
		1.6×10^{-4}	
		2.0×10^{-4}	
		2.1×10^{-4}	
		1.7×10^{-4} [c]	

[a] Orifice area is 0.011 cm^2 and $[BA]_0$ is 4.0×10^{11} molec cm^{-3}.

[b] Uncertainties represent 2σ statistical errors. Overall error includes possible systematic errors estimated to be ± 60% due to uncertainties associated with sample surface areas of the polydisperse powders and the correction to I_0 and I_r from the BA background.

[c] SA mass is 0.2801 g.

Table S4. Uptake coefficients ($\gamma \pm 2\sigma$) for BA on glutaric acid (GA) as a function of BA initial concentration ($[BA]_0$) at 298 K.[a]

$[BA]_0$ (10^{11} molec cm^{-3})	GA mass (g)	GA Surface area (cm^2)	γ	Averaged $\gamma \pm 2\sigma$ [b]
4.0	0.0175 – 0.0943	1.1 – 6.2		0.11 ± 0.04 [c]
10	0.0321	2.1	0.15	0.15 ± 0.02
	0.0617	4.1	0.15	
48	0.0352	2.3	0.17	0.17 ± 0.05
	0.0321	2.1	0.20	
	0.0353	2.3	0.15	
74	0.0259	1.7	0.28	0.25 ± 0.05
	0.0384	2.5	0.26	
	0.0384	2.5	0.23	
1.8×10^2	0.0254	1.7	0.37	0.33 ± 0.11
	0.0353	2.3	0.29	
3.2×10^2	0.0317	2.1	0.44	0.39 ± 0.14
	0.0317	2.1	0.35	

[a] Orifice area is 0.33 cm^2.

[b] Uncertainties represent 2σ statistical errors. Overall error includes possible systematic errors estimated to be ± 60% due to uncertainties associated with sample surface areas of the polydisperse powders and the correction to I_0 and I_r from the BA background.

[c] Averaged from 19 points summarized in Table S2.

References

(1) Franzini, J. B.; Finnemore, E. J.; Daugherty, R. L. *Fluid Mechanics with Engineering Applications*, 9th ed.; McGraw-Hill: New York, 1997.

(2) Fairhurst, M. C.; Ezell, M. J.; Kidd, C.; Lakey, P. S. J.; Shiraiwa, M.; Finlayson-Pitts, B. J. Kinetics, Mechanisms and Ionic Liquids in the Uptake of n-Butylamine onto Low Molecular Weight Dicarboxylic Acids. *Phys. Chem. Chem. Phys.* **2017**, *19* (6), 4827–4839. https://doi.org/10.1039/C6CP08663B.

(3) Koop, T.; Bookhold, J.; Shiraiwa, M.; Pöschl, U. Glass Transition and Phase State of Organic Compounds: Dependency on Molecular Properties and Implications for Secondary Organic Aerosols in the Atmosphere. *Phys. Chem. Chem. Phys.* **2011**, *13* (43), 19238. https://doi.org/10.1039/c1cp22617g.

(4) Knight, C.; Voth, G. A. The Curious Case of the Hydrated Proton. *Acc. Chem. Res.* **2012**, *45* (1), 101–109. https://doi.org/10.1021/ar200140h.

(5) Li, X.; Ogihara, T.; Abe, M.; Nakamura, Y.; Yamago, S. The Effect of Viscosity on the Diffusion and Termination Reaction of Organic Radical Pairs. *Chem. Eur. J.* **2019**, *25* (42), 9846–9850. https://doi.org/10.1002/chem.201902074.
