# Supplementary figures and images for: Distinct Temperature Trends in the Uptake of Gaseous n-Butylamine on Two Solid Diacids
Source: ACS EST Air. 2023 Nov 29;1(1):52–61. doi: 10.1021/acsestair.3c00032 (PMC10798143; doi:10.1021/acsestair.3c00032)

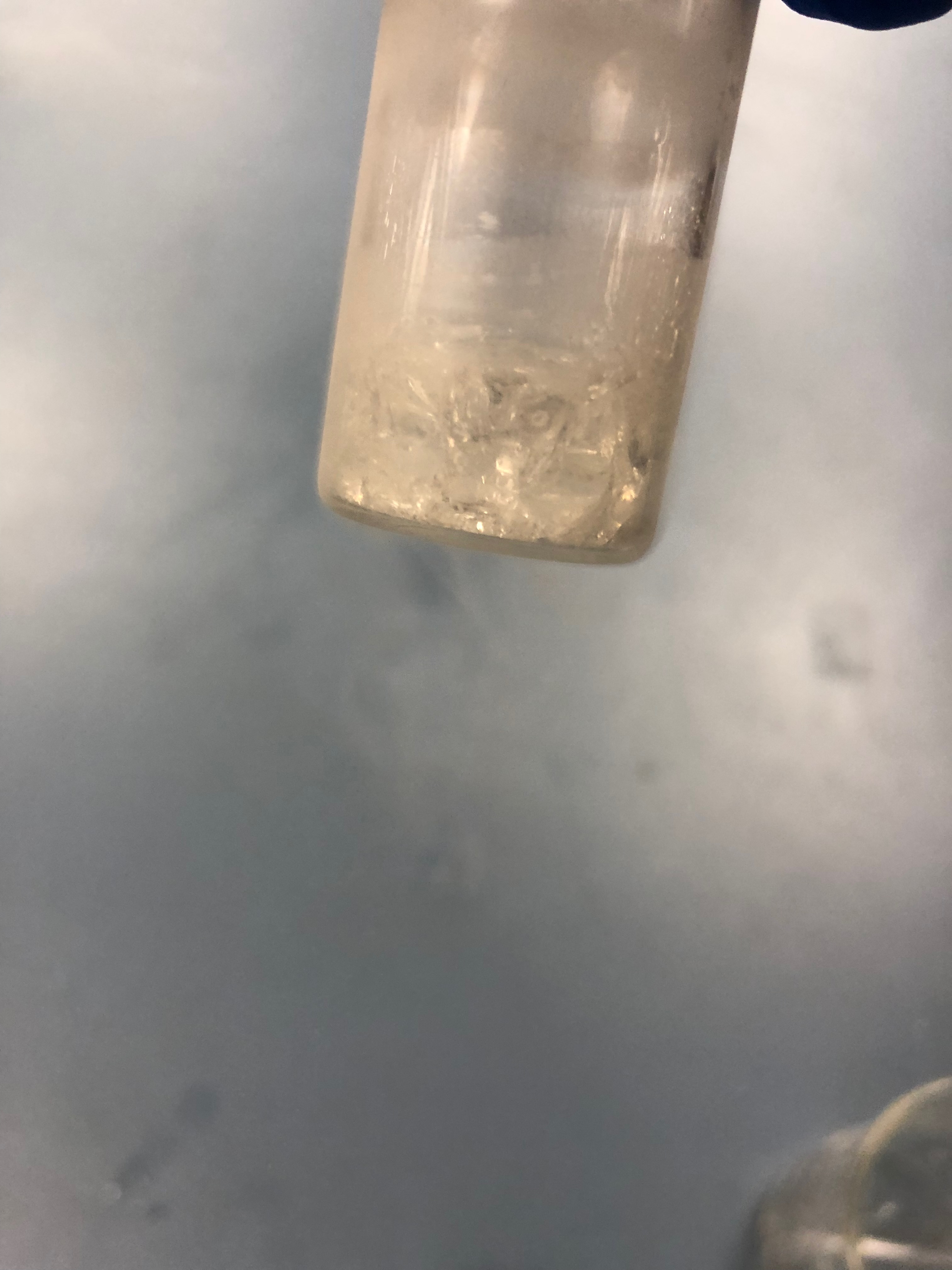

Supplement: Supplementary file 2 — ea3c00032_si_002.zip [file ea3c00032_si_002.zip › viscosity videos/77 K-2:1.jpeg]

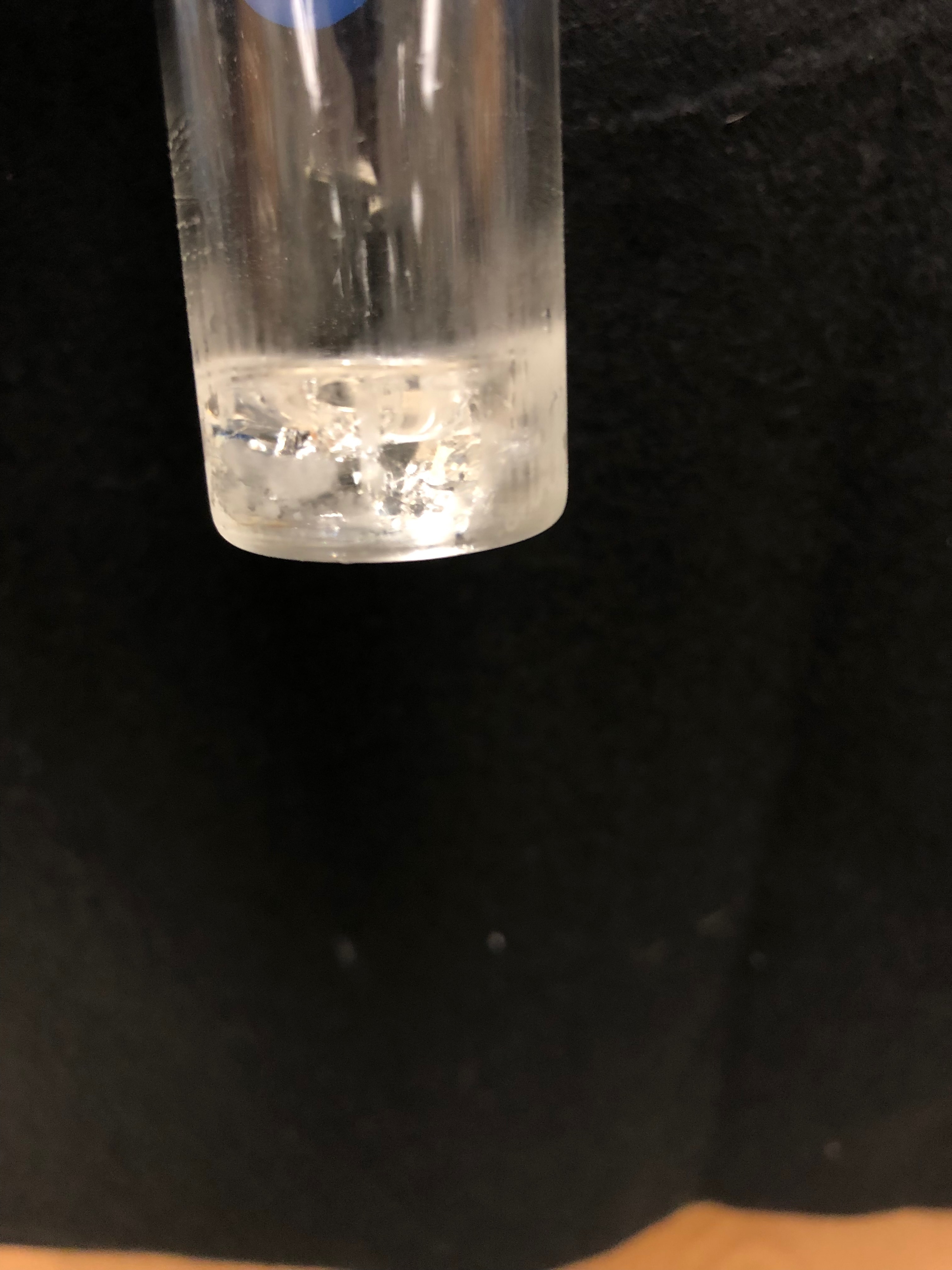

Supplement: Supplementary file 2 — ea3c00032_si_002.zip [file ea3c00032_si_002.zip › viscosity videos/77 K-1:1.jpeg]
